# Malignant *Aspergillus flavus* Otitis Externa with Jugular Thrombosis

**DOI:** 10.3201/eid2504.180710

**Published:** 2019-04

**Authors:** Maxime Moniot, Marion Montava, Stéphane Ranque, Ugo Scemama, Carole Cassagne, Varoquaux Arthur

**Affiliations:** Aix-Marseille University, Marseille, France (M. Moniot, S. Ranque, C. Cassagne);; La Conception University Hospital, Marseille (M. Montava, U. Scemama, V. Arthur)

**Keywords:** *Aspergillus flavus*, jugular thrombosis, magnetic resonance imaging, malignant otitis externa, skull base osteomyelitis, fungal infection, dot in circle sign, diagnostic imaging, voriconazole, ink smudge pattern, fungi, France, ear infection

## Abstract

We report a case of malignant otitis externa with jugular vein thrombosis caused by *Aspergillus flavus*. Magnetic resonance imaging revealed an unusual ink smudge pattern deep in a cervical abscess. The pattern was consistent with mycetoma and may be important for diagnosing these life-threatening infections.

A 73-year-old male patient sought care from the otorhinolaryngology department at University Hospital, Marseille, France. He had a 5-month history of malignant otitis externa (MOE), which was worsening despite 4 months of treatment with intravenous ceftazidime, oral ciprofloxacin, and topical neomycin, polymyxin B, dexamethasone, and thiomersal combination. The patient had a history of high blood pressure, treated with perindopril and nicardipine, and diabetes mellitus, inadequately controlled (hemoglobin A1c 7.7%) with metformin and sitagliptin.

The patient was admitted, and otoscopic examination found otorrhea, inflammation, and stenosis of the right external auditory canal; we could not see the tympanic membrane. Examination of the cranial nerve was normal. Pure-tone audiogram showed a right mixed hearing loss with air-bone gap at 15 dB and symmetric bone curve by presbycusis. Laboratory testing showed elevated erythrocyte sedimentation level (42 mm at 1 h, 82 mm at 2 h) and leukocytosis (11 g/L); C-reactive protein results were within reference range. A computed tomography (CT) scan of the head showed thickening of the ear skin; focal tympanal bone osteolysis; partial right mastoid air cells and middle-ear cavity opacification; and osteolysis of the occipital, styloid, and mastoid bones consistent with MOE (Appendix Figure). Magnetic resonance imaging (MRI) with contrast media confirmed skull base osteomyelitis, evidenced by bone lysis and marrow enhancement of the clivus (Figure, panels A–C). Both MRI and CT showed a right jugular vein thrombosis and cellulitis and abscess in the carotid and perivertebral spaces. Abscess content had an unusual aspect: T2-weighted imaging signal void foci surrounded by a hypersignal rim.

**Figure:**
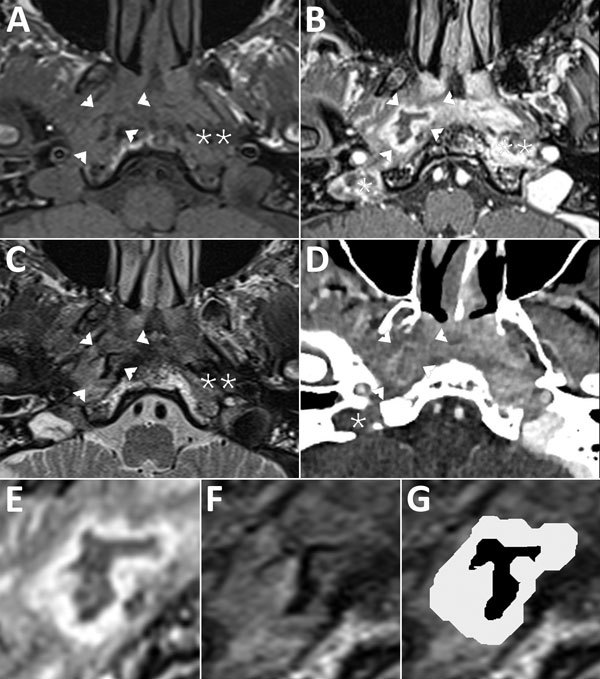
Magnetic resonance imaging (MRI) of a patient with malignant otitis externa, France. Cross-sectional imaging demonstrates a central skull base osteomyelitis in patient’s temporal bone. A) T1-weighted imaging; B, E) 3-dimensional T1-weighted imaging with gadolinium enhancement and fat saturation; C, F, G) T2-weighted imaging; and CT with iodine enhancement (D). Single asterisks (*) indicate jugular bulb thrombosis (panels B, D); double asterisks (**) indicate deep-spaces cellulitis (panels A–C). Arrowheads indicate parapharyngeal abscess at right (panels A–D); parapharyngeal abscess is also visible as a gray layer (panels E, G). The content of the abscess has an unusual “ink smudge” pattern with no signal in T2-weighted imaging, visible as a black layer (panels F, G). This pattern is consistent with a mycetoma surrounded by granulation tissue.

We treated the right jugular vein thrombosis with enoxaparin. The patient underwent surgical debridement with facial nerve monitoring; we collected transmastoid biopsy samples and pus for microbiological analysis and inserted a transtympanic aerator. Direct microscopic examination of the samples showed hyaline septate hyphae consistent with hyalohyphomycosis. Biopsy samples grew 2 bacteria, *Corynebacterium striatum* and *Enterococcus faecalis,* and 1 filamentous fungus, *Aspergillus flavus*, that we identified by matrix-assisted laser desorption/ionization time-of-flight mass spectrometry (Microflex LT, https://www.bruker.com) against an in-house database described by Normand et al*.* (*1*). Etest antifungal susceptibility testing (bioMérieux, https://www.biomerieux.com) showed that the *A. flavus* strain was sensitive to voriconazole (MIC 0.380 mg/L) and resistant to amphotericin B (MIC 12 mg/L). We stopped administration of auricular drops, continued intravenous ceftazidime (1.5 g/d) and oral ciprofloxacin (1.5 g/d), and started voriconazole therapy (6 mg/kg/12 h intravenously, followed by 400 mg/d orally). Otalgia, otorrhea, and inflammatory external auditory canal symptoms were relieved, and the patient recovered after 6 weeks. No further follow-up was available.

Fungi cause ≈10% of MOE (*2*). The 3 leading species, by decreasing frequency, are *A. fumigatus*, *A. flavus*, and *A. niger* (*3*). *A. flavus* is more frequently involved in MOE than is *A. niger* (*3*,*4*).

Jugular vein thrombosis (JVT) was previously reported in MOE (*5*) and other conditions such as Lemierre syndrome, invasive fungal infection, or any inflammatory process including otitis media. Various pathogens can cause JVT, especially *Fusobacterium necrophorum* and zygomycetes. Data on JVT in MOE are scarce; we could find no previously reported case of JVT related to *A. flavus* MOE. Postcontrast CT with soft tissue algorithm is considered the first-line imaging modality to diagnose JVT (*5*). In our case, both CT and MRI confirmed the diagnosis.

Osteomyelitis and abscess showed on MRI but were hardly visible on CT (Appendix Figure). High-signal T2-weighted imaging is typical in purulent content of abscesses (*6*). In contrast, this case exhibited an unusual lack of T2-weighted imaging signal. This characteristic pattern is known in various mycetoma locations as paranasal fungus balls (*7*) or maduromycosis, for which the T2-weighted imaging dot-in-circle sign is specific (*8*). Most authors explain the signal void as a magnetic susceptibility behavior on T2-weighted imaging resulting from accumulation of iron and other magnetic atoms (*9*). This case introduced a new T2-weighted imaging signal void pattern we refer to as an “ink smudge” appearance. A bone sequestrum is a differential diagnosis, yet this lesion lacked calcification in CT. We hypothesize that the ink-smudge sign we identified could be specific to fungal infection. This report should prompt careful assessment by MRI of deep-space abscess in patients with MOE.

The standard treatment for fungal MOE is a combination of surgical debridement, systemic antifungal therapy, and control of concurrent conditions. There is no consensus for the duration of the antifungal treatment; patients usually receive 6–8 of weeks antifungal therapy, more if clinical examination or imaging follow-up supports extending treatment (*10*). 

We highlight the potential use of an MRI ink-smudge pattern to identify fungal infection in MOE. Furthermore, because we saw JVT on both postcontrast CT and MRI scans, our findings and these images may be crucial for improving patient prognosis through timely and adequate treatment.

Appendix Additional information about malignant *Aspergillus flavus* otitis externa with jugular thrombosis.
