# Supplementary material for: Malignant Aspergillus flavus Otitis Externa with Jugular Thrombosis
Source: Emerg Infect Dis. 2019 Apr;25(4):830–2. doi: 10.3201/eid2504.180710 (PMC6433013; doi:10.3201/eid2504.180710)
Supplement: Appendix — Additional information about malignant Aspergillus flavus otitis externa with jugular thrombosis. [file 18-0710-Techapp-s1.pdf]

Article DOI: https://doi.org/10.3201/eid2504.180710

Malignant *Aspergillus flavus* Otitis Externa with Jugular Thrombosis

Appendix

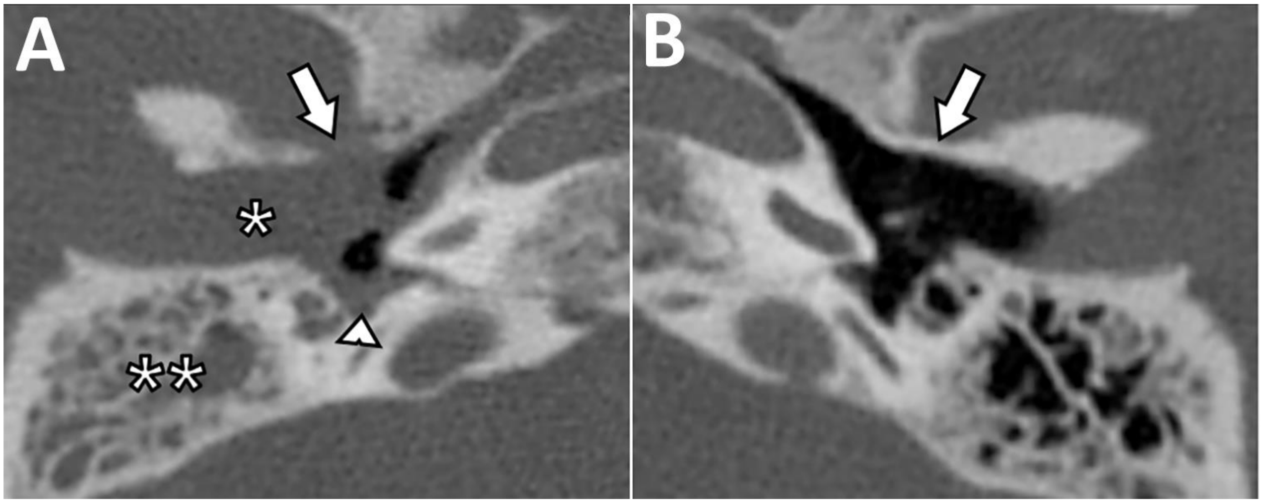

Appendix Figure. Normal malignant otitis externa features. Temporal bone image from computed tomography, performed without contrast media, in bone reconstruction algorithm and axial planes. A) Patient's right ear, with the usual features of malignant otitis externa: cutaneous thickening of the external ear canal (*) against which tympanal bone osteolysis is visible, close to petrotympanic fissure (arrow). Inflammatory changes in the middle ear cavity consist of a mucosal thickening (arrowhead) and filling of mastoid cell (**). B) Patient's left ear, with normal and continuous tympanal bone (arrow).
